# Tetra­nuclear copper(II) complex of 2-hydroxy-*N*,*N*′-bis­[1-(2-hy­droxy­phen­yl)ethyl­idene]­propane-1,3-di­amine

**DOI:** 10.1107/S2056989022002225

**Published:** 2022-03-01

**Authors:** Alassane Saïdou Diallo, Ibrahima Elhadji Thiam, Mbossé Gueye-Ndiaye, Moussa Dieng, James Orton, Coles Simon, Mohamed Gaye

**Affiliations:** a Département de Chimie, UFR SATIC, Université Alioune Diop, Bambey, Senegal; b Département de Chimie, Faculté des Sciences et Techniques, Université Cheik Anta Diop, Dakar, Senegal; c UK National Crystallography Service, School of Chemistry, Faculty of Engineering and Physical Sciences, University of Southampton, SO17 1BJ, UK

**Keywords:** crystal structure, 1-(2-hy­droxy­phen­yl)ethanone, 1,3-di­amino­propan-2-ol

## Abstract

The title mol­ecular structure, namely, (μ_3_-acetato)(μ_2_-acetato)­bis­(μ_3_-1,3-bis­{[1-(2-oxidophen­yl)ethyl­idene]amino}­propan-2-olato)tetra­copper(II) monohydrate, [Cu_4_(C_19_H_19_N_2_O_3_)_2_(CH_3_CO_2_)_2_]·H_2_O, corresponds to a non-symmetric tetra­nuclear copper complex. The complex exhibits one ligand mol­ecule that connects two copper Cu^II^ metal centres *via* its ethano­lato oxygen anion acting in a μ_2_-mode and one ligand mol­ecule that connects three copper Cu^II^ metal centres *via* its ethano­lato oxygen anion acting in a μ_3_-mode. One bridging acetate group acting in an η^1^:η^1^-μ_2_-mode connects two copper(II) ions while another bridging acetate group connects three copper(II) ions in an η^1^:-η^2^-μ_3_-mode. A chair-like Cu_3_O_3_ structure is generated in which the two CuO_4_N units are connected by one μ_2_-O ethano­late oxygen atom. These two units are connected respectively to the CuO_3_N unit *via* one μ_3_-O ethano­late oxygen atom and one μ_2_-O atom from an acetate group. The μ_3_-O atom also connects one of the CuO_4_N units and the CuO_3_N unit to another CuO_3_N unit, which is out of the chair-like structure. Each of the two penta­coordinated Cu^II^ cations has a distorted NO_4_ square-pyramidal environment. The geometry of each of the two CuNO_3_ units is best described as a slightly square-planar environment. A series of intra­molecular O—H⋯O hydrogen bonds is observed. In the crystal, the units are connected by inter­molecular C—H⋯O and O—H⋯O hydrogen bonds, thus forming sheets parallel to the *ac* plane

## Abstract 2

In the title Schiff base tetra­nuclear copper(II) complex, two discrete environments are present in the structure: CuNO_4_ and CuNO_3_. Two copper(II) cations are situated in distorted square-pyramidal environment, while two copper(II) cations are located in a slightly square-planar geometry. One bridging acetate group acting in an η^1^:η^1^-μ_2_-mode connects two copper(II) ions, while another bridging acetate group connects three copper(II) ions in an η^1^:-η^2^–μ_3_-mode.

## Chemical context

**Figure 1:**
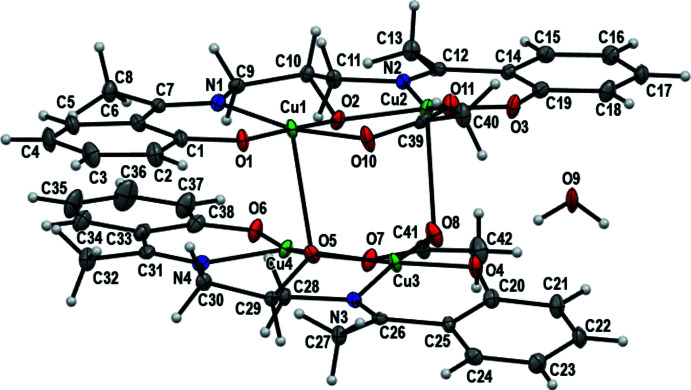
A view of the title compound, showing the atom-numbering scheme.

The controlled design of new coordination complexes of transition metals from polydentate ligands is of great inter­est for research, because of the potential applications that these functional materials can have and for their inter­esting structural diversity (Popov *et al.*, 2012 ▸; Mitra *et al.*, 2014 ▸). In this context, important research is being devoted to the chemistry of transition-metal complexes with different oxidation states incorporating polydentate ligands with N and O donor sites (Xie *et al.*, 2012 ▸; Banerjee & Chattopadhyay, 2019 ▸; Ferguson *et al.*, 2006 ▸). These ligands can act in a versatile manner and generate compounds with very different structures, depending on the metal–ligand ratio and the nature of the metal cation (Fernandes *et al.*, 2000 ▸). In this context, penta­dentate Schiff bases have made it possible to synthesize several complexes with various transition-metal cations, resulting in an unusual coordination environment with inter­esting stereochemistry (Banerjee *et al.*, 2011 ▸). Depending on the size of the cation and its external electronic configuration and the flexibility of the ligand, novel structures with high nuclearity have been obtained (Aly, 1999 ▸). These compounds are very attractive for the above reasons, and they have been widely used in several studies. Many multinuclear transition-metal complexes with various structures have been generated, depending on the disposition of the metal ions and donor sites (N or O). Tetra­nuclear (Asadi *et al.*, 2018 ▸; Manna *et al.*, 2019 ▸), penta­nuclear (Hari *et al.*, 2019 ▸; Ghosh, Clérac *et al.*, 2013 ▸) hexa­nuclear (Shit *et al.*, 2013 ▸; Kébé *et al.*, 2021 ▸) and hepta­nuclear (Gheorghe *et al.*, 2019 ▸; Ghosh, Bauzá *et al.*, 2013 ▸) forms have reported with potential applications in the fields of magnetism (Gheorghe *et al.*, 2019 ▸), catalysis (Nesterova *et al.*, 2020 ▸; Das *et al.*, 2018 ▸) or biomimetic synthesis (Nesterova *et al.*, 2020 ▸; Sanyal *et al.*, 2017 ▸). Our research group has already enabled us to prepare several multidentate Schiff base complexes (Mamour *et al.*, 2018 ▸; Sarr *et al.*, 2018*a* ▸,*b* ▸; Sall *et al.*, 2019 ▸). We then explored the possibility of preparing complexes with several metal cations from a penta­dentate Schiff base obtained by condensation of 1,3-di­amino­propan-2-ol and 1-(2-hy­droxy­phen­yl)ethanone, which is rich in hydroxyl groups. From this Schiff base we prepared a hexa­nuclear complex with an open-cube structure (Kébé *et al.*, 2021 ▸). In a continuation of our work with this Schiff base, we obtained the title tetra­nuclear copper complex (Fig. 1 ▸) whose structure is presented herein. 
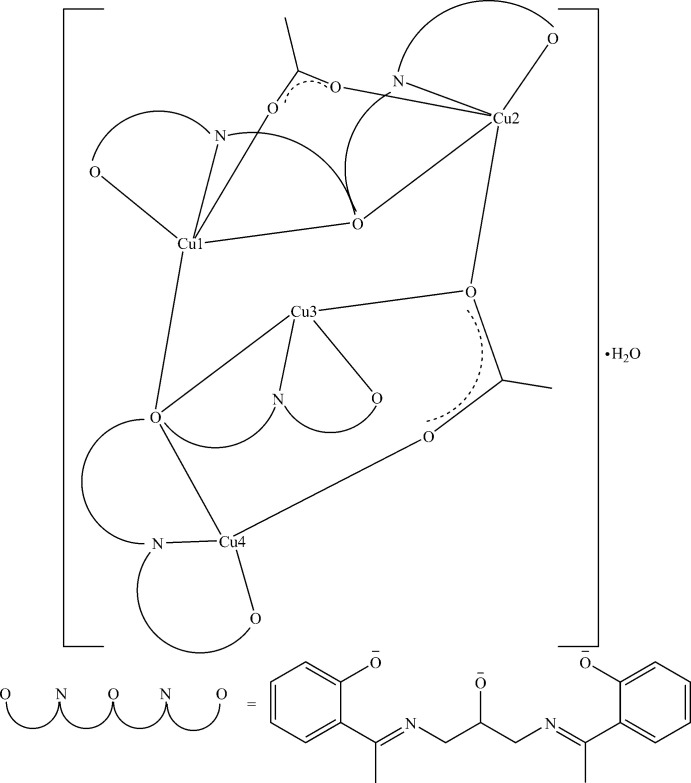


## Structural commentary

*N*,*N*′-Bis­{[1-(2-hy­droxy­phen­yl)ethyl­idene)]}-2-hy­droxy­pro­pane-1, 3-di­amine (H_3_ *L* was synthesized *via* a condensation reaction between 1,3-di­amino­propan-2-ol and 1-(2-hy­droxy­phen­yl)ethanone in a 1:2 ratio in ethanol. Mixing H_3_ *L* and hydrated copper acetate yielded a tetra­nuclear complex formulated as [Cu_4_ *L*_2_(CH_3_CO_2_)_2_]·H_2_O in which the ligand acts in its tri-deprotonated *L^−3^* form. In the tetra­nuclear complex, one of the *L^−3^* anions acts in μ_2_-mode, connecting the two penta­coordinated Cu^II^ cations. The second *L^−3^* anion acts in μ_3_ mode, connecting the two tetra­coordinated Cu^II^ cations and one of the penta­coordinated Cu^II^ cations. The second penta­coordinated Cu^II^ cation is connected to the two tetra­coordinated Cu^II^ cations *via* an acetate group acting in η^1^:η^2^-μ_3_ mode. Additionally, the two penta­coordinated Cu^II^ cations are connected by an acetate group acting in η^1^:η^1^-μ_2_ mode. For each ligand, the azomethine nitro­gen atom and the phenolate oxygen atom of one arm are both linked to one Cu^II^ cation while the corresponding atoms of the other arm are bonded to another Cu^II^ cation. No phenolate oxygen atom acts in bridging mode. In one ligand the ethano­late oxygen atom bridges the two penta­coordinated Cu^II^ cations, and in the second ligand the ethano­late oxygen atom bridges the two tetra­coordinated Cu^II^ cations and one penta­coordinated Cu^II^ cation. The two *L^−3^* ligands are coordinated differently in hexa­dentate (-η^1^-*O*_phenolate_, -η^1^-*N*_imino_, -μ_2_-*O*_enolato_, -η^1^-*N*_imino_, -η^1^-*O*_phenolato_) and hepta­dentate (-η^1^-*O*_phenolate_, -η^1^-*N*_imino_, -μ_3_-*O*_enolato_, -η^1^-*N*_imino_, -η^1^-*O*_phenolato_) fashions. Four five-membered CuOCCN rings and four six-membered CuOCCCN rings are formed upon the coordination of the ligand mol­ecules. In the tetra­nuclear complex, two discrete CuO_4_N and CuO_3_N units are observed.

Atoms Cu1 and Cu2 are penta­coordinated and their environments can be best described as slightly distorted square-pyramidal. The Addison *τ* parameter (Addison *et al.*, 1984 ▸) calculated from the largest angles (Table 1 ▸; *τ* = 0 for perfect square-pyramidal and *τ* = 1 for perfect trigonal–bipyramidal geometries, respectively) around the metal ion are *τ* = 0.1103 for Cu1 and *τ* = 0.1887 for Cu2. For Cu1 and Cu2, the basal planes are occupied by one phenolate oxygen anion, one azomethine nitro­gen atom, one ethano­late oxygen atom and one oxygen atom from the η^1^:η^1^-μ_2_ acetate group, the apical position being occupied by an ethano­late oxygen atom from a second ligand mol­ecule for Cu1 and an oxygen atom from the η^1^:η^2^-μ_3_ acetate group for Cu2. The atoms forming the basal plane for Cu1 (N1, O1, O2, O10) are almost coplanar (r.m.s. deviation = 0.1088 Å) and the Cu1 atom is displaced toward the O5 atom, which occupies the apical position, by 0.0545 (2) Å. The Cu1—O5 distance of 2.749 (3) Å is longer than the distances between Cu1 and the atoms in the basal plane [Cu1—N_ligand_ = 1.966 (4) Å, Cu1—O_ligand_ = 1.878 (3) and 1.916 (3) Å and Cu1—O_acetate_ = 1.982 (3) Å)], as expected for a Jahn–Teller distortion (Monfared *et al.*, 2009 ▸), typical of a Cu^II^ *d* ^9^ configuration (Monfared *et al.*, 2009 ▸). These values are in accordance with those in similar copper(II) complexes (Haldar *et al.*, 2016 ▸; Siluvai & Murthy, 2009 ▸). The *cisoid* and *transoid* angles are in the ranges 85.01 (14)–95.10 (14)° and 169.71 (16)–176.33 (14)°, respectively. The atoms forming the basal plane for Cu2 (N2, O2, O11, O3) are less coplanar than those around Cu1 (r.m.s. deviation = 0.2086 Å) and the Cu2 atom is displaced toward the O8 atom, which occupies the apical position, by 0.0808 (1) Å. The from Cu2—O8 distance of 2.703 (4) Å is longer than those to atoms in the equatorial plane [Cu2—N_ligand_ = 1.961 (4) Å, Cu2—O_ligand_ = 1.877 (3) and 1.920 (3) Å and Cu2—O_acetate_ = 1.940 (3) Å]. As observed for Cu1, Jahn–Teller distortion (Monfared *et al.*, 2009 ▸) is responsible of the elongation of the distance between Cu2 and the apical atom O8. The *cisoid* and *transoid* angles are in the ranges 85.74 (15)–96.89 (14)° and 161.66 (15)–173.00 (15)°, respectively. The bond lengths involving the μ_2_-bridging ethano­lato oxygen atom and the copper cations are asymmetrical: Cu1—O2 = 1.916 (3) Å and Cu2—O2 = 1.920 (3) Å. The distances between the μ_3_-bridging ethano­lato oxygen atom and the copper cations are very different: Cu1—O5 = 2.749 (3) Å, Cu3—O5 = 1.907 (3) Å and Cu4—O5 = 1.921 (3) Å. The copper cations Cu3 and Cu4 are coordinated by one ethano­lato oxygen anion, one phenoxo oxygen anion, one azomethine nitro­gen atom of the ligand and one oxygen atom of a η^1^:η^2^-μ_3_ acetate group (O8 for Cu3 and O7 for Cu4). The Cu3—O4 [1.873 (3) Å], Cu3—O5 [1.907 (3) Å], Cu3—N3 [1.947 (4) Å], Cu3—O8 [1.957 (3) Å], Cu4—O6 [1.869 (3) Å], Cu4—O5 [1.921 (3) Å], Cu4—N4 [1.962 (4) Å] and Cu4—O7 [1.955 (3) Å] distances are in close proximity to values reported for copper(II) complexes with analogous Schiff base ligands (Patra *et al.*, 2015 ▸; Lukov *et al.*, 2017 ▸). For the Cu3 and Cu4 centres, the coordination environment can be best described as distorted square planar with r.m.s. deviations of 0.7870 Å for N3/O4/O8/O5/Cu3 and 0.7921 Å for O5/O7/O6/N4/Cu4. These planes, which share one vertex (O5), form a dihedral angle of 65.67 (1)°. The tetra­gonality parameter (Singh *et al.*, 2017 ▸) *τ_4_* values of 0.0993 (Cu3) and 0.1801 (Cu4) suggested distorted square-planar geometries. For the two copper cations the *cisoid* angles are in the ranges 86.17 (14)–93.29 (15)° for Cu3 and 84.04 (14)–96.93 (14)° for Cu4 and the *transoid* angles are O4—Cu3—O5 = 177.07 (15)°, O8—Cu3—N3 = 173.28 (15)°, O6—Cu4—O5 = 170.48 (14)° and O7—Cu3—N4 = 164.11 (15)°. The C—N bonds are in the range 1.291 (6)–1.300 (6) Å, indicative of double-bond character and the presence of the imino groups in the two ligands.

## Supra­molecular features

**Figure 2:**
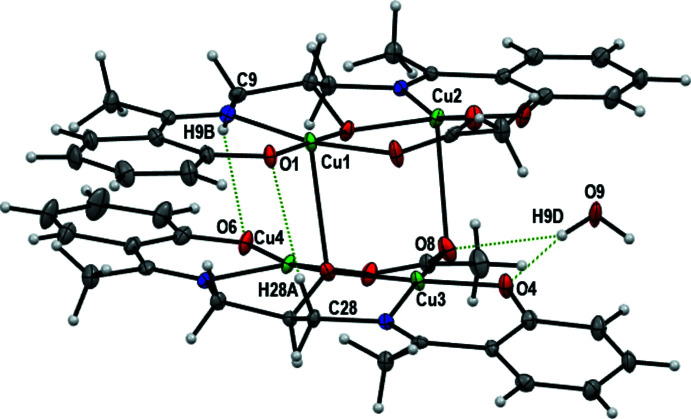
Detail of the structure of the complex showing the O—H⋯O and C—H⋯O hydrogen bonds.

**Figure 3:**
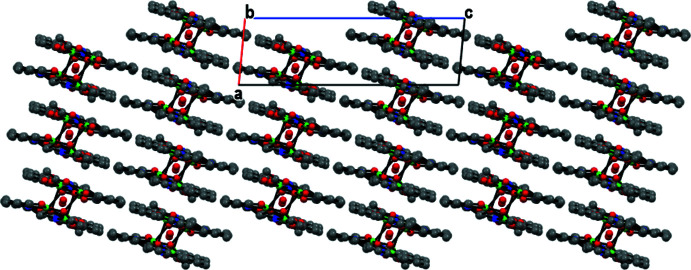
Sheets parallel to the *ac* plane.

**Figure 4:**
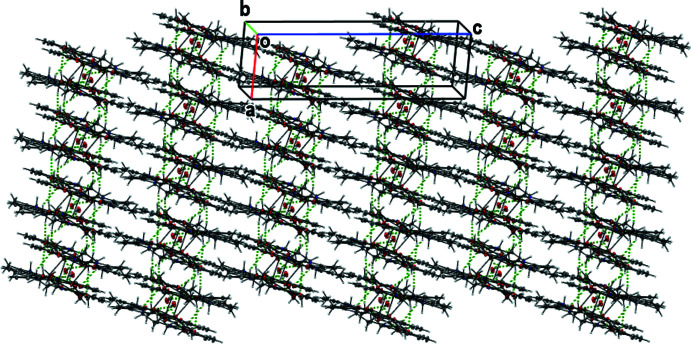
View of the two-dimensional sheets parallel to the *ac* plane.

Intra­molecular O—H⋯O hydrogen bonds involving the uncoordinated water mol­ecule, a phenoxo oxygen atom and an oxygen atom of acetate group and C—H⋯O_phenoxo_ are observed (Fig. 2 ▸, Table 2 ▸). The uncoordinated water mol­ecule is situated into the void of the tetra­nuclear complex and has O⋯O contacts of 2.894 (5) and 3.158 (5) Å suggesting medium-strength hydrogen bonds. In the crystal, the complex mol­ecules are arranged in sheets parallel to the *ac* plane (Fig. 3 ▸). The sheets are connected by C—H⋯O bonds (C—H⋯O_phenoxo_, C—H⋯O_water_, C—H⋯O_acetate_; Table 2 ▸). The series of inter­molecular and intra­molecular hydrogen bonds stabilize and link the components into two-dimensional sheets parallel to the *ac* plane (Fig. 4 ▸).

## Database survey

*N*,*N*′–Bis[(1-(2-hy­droxy­phen­yl)ethyl­idene)]-2-hy­droxy­pro­pane-1,3-di­amine is widely used in coordination chemistry. The current release of the CSD (Version 5.42, November 2021 update; Groom *et al.*, 2016 ▸) gave eleven hits. Three are complexes of the ligand with Ni^II^ cations [KARPOK and KARPUQ (Liu *et al.*, 2012 ▸); OMOFUS (Banerjee *et al.*, 2011 ▸)]. Four entries are complexes of Cu^II^ cations [KUKTAM (Basak *et al.*, 2009 ▸), NADDIJ and NADDOP (Osypiuk *et al.*, 2020 ▸), OVOWAA (Kébé *et al.*, 2021 ▸)]. In addition, two Co^II^ complexes (OMOFOM and OMOGAZ; Banerjee *et al.*, 2011 ▸), one Fe^II^ (RIDHUJ; Biswas *et al.*, 2013 ▸) and one V^V^ complex (KEWGUQ; Maurya *et al.*, 2013 ▸) have been reported. In all eleven cases, the ligand acts in a penta­dentate mode through the two soft azomethine nitro­gen atoms, the two hard phenolate oxygen anions and the one hard enolate oxygen anion. In seven cases (KARPOK, KARPUQ, OMOFUS, KUKTAM, NADDIJ, NADDOP and OMOGAZ), the complexes are tetra­nuclear while two dinuclear (OMOFOM and RIDHUJ), one mononuclear (KEWGUQ) and one hexa­nuclear (OVOWAA) complex have been reported.

## Synthesis and crystallization

The ligand *N,N* ^’^-bis­[(1-(2-hy­droxy­phen­yl)ethyl­idene)]-2-hy­droxy­propane-1,3-di­amine (H*L*_3_) was prepared from 1-(2-hy­droxy­phen­yl)ethanone and 2-hy­droxy­propane-1,3-di­amine in a 2:1 ratio in ethanol according to a slight modification of a literature method (Song *et al.*, 2003 ▸). To a solution of 1,3-di­amino­propane-2-ol (0.900 g, 10 mmol) in 25 mL of ethanol was added dropwise (2-hy­droxy­phen­yl)ethanone (2.720 g, 20 mmol). The resulting orange mixture was refluxed for 3 h, affording the organic ligand H_3_ *L*. On cooling, the yellow precipitate that appeared was recovered by filtration and dried in air. Yield 75%. m.p. 479–480 K. FT–IR (KBr, ν, cm^−1^): 3538 (OH), 3268 (OH), 1605 (C=N), 1538 (C=C), 1528 (C=C), 1455 (C=C), 1247 (C—O), 1043, 760. Analysis calculated for C_19_H_22_N_2_O_3_: C, 69.92; H, 6.79; N, 8.58. Found: C, 69.90; H, 6.76; N, 8.56%.

A solution of Cu(CH_3_CO_2_)_2_·(H_2_O) (0.1996 g, 1 mmol) in 5 mL of ethanol was added to a solution of H_3_ *L* (0.163 g, 0.5 mmol) in 10 mL of ethanol at room temperature. The initial yellow solution immediately turned deep green and was stirred for 30 min before being filtered. The filtrate was kept at 298 K. After one week, light-green crystals suitable for X-ray diffraction were collected and formulated as [Cu_4_ *L*_2_(CH_3_CO_2_)_2_]·H_2_O. FT–IR (KBr, ν, cm^−1^): 3404, 1601, 1532, 1332, 1299, 895, 760. Analysis calculated for C_42_H_46_Cu_4_N_4_O_11_: C, 48.64; H, 4.47; N, 5.40. Found: C, 48.60; H, 4.49; N, 5.44%.

## Refinement

Crystal data, data collection and structure refinement details are summarized in Table 3 ▸. H atoms attached to the hydroxyl group and water mol­ecules were located in a difference-Fourier map and freely refined. Other H atoms (CH, CH_2_, CH_3_ groups and hydroxyl of ethanol mol­ecules) were geometrically optimized (O—H = 0.85 Å, C—H = 0.93–0.97 Å) and refined using a riding model (AFIX instructions) with *U*_iso_(H) = 1.2*U*_eq_(C) or 1.5*U*_eq_(C) for CH_3_ and OH groups.

## Supplementary Material

Crystal structure: contains datablock(s) I. DOI: 10.1107/S2056989022002225/ex2053sup1.cif

Structure factors: contains datablock(s) I. DOI: 10.1107/S2056989022002225/ex2053Isup3.hkl

CCDC reference: 2154581

Additional supporting information:  crystallographic information; 3D view; checkCIF report

## Figures and Tables

**Table 1:** Selected geometric parameters (Å, °)

Cu2—O2	1.920 (3)	Cu1—N1	1.966 (4)
Cu2—O3	1.877 (3)	Cu3—O5	1.907 (3)
Cu2—O11	1.940 (3)	Cu3—O4	1.873 (3)
Cu2—O8	2.703 (4)	Cu3—O8	1.957 (3)
Cu2—N2	1.961 (4)	Cu3—N3	1.947 (4)
Cu1—O5	2.749 (3)	Cu4—O5	1.921 (3)
Cu1—O2	1.916 (3)	Cu4—O7	1.955 (3)
Cu1—O10	1.982 (3)	Cu4—O6	1.869 (3)
Cu1—O1	1.878 (3)	Cu4—N4	1.962 (4)
			
O3—Cu2—O2	173.00 (15)	O4—Cu3—O5	177.07 (15)
O11—Cu2—N2	161.66 (15)	N3—Cu3—O8	173.28 (15)
O1—Cu1—O2	176.33 (14)	O7—Cu4—N4	164.11 (15)
N1—Cu1—O10	169.71 (16)		

**Table 2:** Hydrogen-bond geometry (Å, °)

*D*—H⋯*A*	*D*—H	H⋯*A*	*D*⋯*A*	*D*—H⋯*A*
O9—H9*C*⋯O4	0.85	2.08	2.894 (5)	159
O9—H9*C*⋯O8	0.85	2.56	3.158 (5)	128
O9—H9*D*⋯O3	0.85	2.08	2.928 (5)	175
C28—H28*A*⋯O1	0.97	2.58	3.427 (6)	146
C29—H29⋯O1^i^	0.98	2.60	3.424 (5)	142
C10—H10⋯O6^ii^	0.98	2.51	3.351 (6)	144
C8—H8*A*⋯O9^iii^	0.96	2.44	3.372 (6)	163
C9—H9*B*⋯O6	0.97	2.65	3.521 (6)	150
C32—H32*A*⋯O9^iii^	0.96	2.38	3.304 (6)	162
C42—H42*A*⋯O11^i^	0.96	2.66	3.256 (7)	121

Symmetry codes: (i) 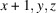 ; (ii) 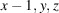 ; (iii) 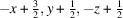 .

**Table 3:** Experimental details

Crystal data	
Chemical formula	[Cu_4_(C_19_H_19_N_2_O_3_)_2_(C_2_H_3_O_2_)_2_]·H_2_O
*M*_r_	1037.02
Crystal system, space group	Monoclinic, *P*2_1_/*n*
Temperature (K)	293
*a*, *b*, *c* (Å)	6.9688 (1), 25.8066 (4), 22.8290 (4)
β (°)	95.418 (2)
*V* (Å^3^)	4087.25 (11)
*Z*	4
Radiation type	Mo *K*α
μ (mm^−1^)	2.12
Crystal size (mm)	0.25 × 0.2 × 0.1

Data collection	
Diffractometer	Nonius KappaCCD
Absorption correction	Multi-scan (*SADABS*; Krause *et al.*, 2015 ▸)
*T*_min_, *T*_max_	0.967, 1.000
No. of measured, independent and observed [*I* > 2σ(*I*)] reflections	12039, 12039, 10024
*R*_int_	0.008
(sin θ/λ)_max_ (Å^−1^)	0.651

Refinement	
*R*[*F* ^2^ > 2σ(*F* ^2^)], *wR*(*F* ^2^), *S*	0.056, 0.131, 1.13
No. of reflections	12039
No. of parameters	560
H-atom treatment	H-atom parameters constrained
Δρ_max_, Δρ_min_ (e Å^−3^)	1.69, −0.88

Computer programs: *APEX3* and *SAINT* (Bruker, 2016 ▸), *SHELXT* (Sheldrick, 2015*a* ▸), *SHELXL2018/3* (Sheldrick, 2015*b* ▸) and *OLEX2* (Dolomanov *et al.*, 2009 ▸).

## supplementary crystallographic information

### Crystal data

**Table:**

[Cu_4_(C_19_H_19_N_2_O_3_)_2_(C_2_H_3_O_2_)_2_]·H_2_O	*F*(000) = 2120
*M**_r_* = 1037.02	*D*_x_ = 1.685 Mg m^−^^3^
Monoclinic, *P*2_1_/*n*	Mo *K*α radiation, λ = 0.71075 Å
*a* = 6.9688 (1) Å	Cell parameters from 5800 reflections
*b* = 25.8066 (4) Å	θ = 2.4–28.7°
*c* = 22.8290 (4) Å	µ = 2.12 mm^−^^1^
β = 95.418 (2)°	*T* = 293 K
*V* = 4087.25 (11) Å^3^	Prismatic, light-green
*Z* = 4	0.25 × 0.2 × 0.1 mm

### Data collection

**Table:**

Nonius KappaCCD diffractometer	10024 reflections with *I* > 2σ(*I*)
CCD scans	*R*_int_ = 0.008
Absorption correction: multi-scan (SADABS; Krause *et al.*, 2015)	θ_max_ = 27.6°, θ_min_ = 1.8°
*T*_min_ = 0.967, *T*_max_ = 1.000	*h* = −9→9
12039 measured reflections	*k* = −33→33
12039 independent reflections	*l* = −29→28

### Refinement

**Table:**

Refinement on *F*^2^	0 restraints
Least-squares matrix: full	Hydrogen site location: mixed
*R*[*F*^2^ > 2σ(*F*^2^)] = 0.056	H-atom parameters constrained
*wR*(*F*^2^) = 0.131	*w* = 1/[σ^2^(*F*_o_^2^) + (0.038*P*)^2^ + 21.6332*P*] where *P* = (*F*_o_^2^ + 2*F*_c_^2^)/3
*S* = 1.13	(Δ/σ)_max_ = 0.001
12039 reflections	Δρ_max_ = 1.69 e Å^−^^3^
560 parameters	Δρ_min_ = −0.88 e Å^−^^3^

### Special details

**Table:**

Geometry. All esds (except the esd in the dihedral angle between two l.s. planes) are estimated using the full covariance matrix. The cell esds are taken into account individually in the estimation of esds in distances, angles and torsion angles; correlations between esds in cell parameters are only used when they are defined by crystal symmetry. An approximate (isotropic) treatment of cell esds is used for estimating esds involving l.s. planes.
Refinement. Refined as a 2-component twin.

### Fractional atomic coordinates and isotropic or equivalent isotropic displacement parameters (Å^2^)

**Table:**

	*x*	*y*	*z*	*U*_iso_*/*U*_eq_	
Cu2	0.60863 (8)	0.28366 (2)	0.32611 (2)	0.01219 (13)	
Cu1	0.52144 (8)	0.38573 (2)	0.22819 (2)	0.01203 (13)	
Cu3	0.84495 (8)	0.29191 (2)	0.17735 (2)	0.01246 (13)	
Cu4	1.01850 (8)	0.39019 (2)	0.27079 (2)	0.01232 (13)	
O5	0.8865 (4)	0.36243 (12)	0.20000 (13)	0.0132 (6)	
O2	0.6231 (5)	0.35435 (12)	0.30062 (14)	0.0140 (6)	
O10	0.4526 (5)	0.31935 (13)	0.18788 (15)	0.0222 (8)	
O7	1.1025 (5)	0.32394 (12)	0.30599 (15)	0.0181 (7)	
O3	0.6275 (5)	0.21453 (12)	0.35156 (15)	0.0185 (7)	
O4	0.7906 (5)	0.22334 (12)	0.15452 (14)	0.0181 (7)	
O1	0.4344 (5)	0.41970 (12)	0.15789 (14)	0.0152 (7)	
O6	1.1072 (5)	0.42189 (12)	0.34184 (15)	0.0180 (7)	
O11	0.4548 (5)	0.25787 (13)	0.25696 (15)	0.0202 (7)	
O8	0.9004 (5)	0.26730 (13)	0.25825 (15)	0.0208 (7)	
N1	0.5427 (5)	0.45103 (14)	0.27266 (17)	0.0126 (8)	
C41	1.0334 (7)	0.27956 (17)	0.2974 (2)	0.0137 (9)	
N3	0.8224 (5)	0.31721 (14)	0.09664 (16)	0.0111 (7)	
O9	0.7291 (6)	0.15395 (13)	0.25055 (17)	0.0264 (8)	
H9C	0.770732	0.177114	0.228491	0.040*	
H9D	0.703080	0.170249	0.281168	0.040*	
N2	0.6827 (5)	0.31062 (14)	0.40532 (16)	0.0114 (7)	
N4	1.0044 (5)	0.45572 (14)	0.22705 (16)	0.0121 (7)	
C39	0.4116 (6)	0.27589 (17)	0.2067 (2)	0.0143 (9)	
C19	0.6706 (6)	0.19719 (18)	0.4053 (2)	0.0135 (9)	
C26	0.7822 (6)	0.29142 (17)	0.04858 (19)	0.0110 (8)	
C16	0.7556 (7)	0.15045 (19)	0.5168 (2)	0.0180 (10)	
H16	0.781948	0.135243	0.553591	0.022*	
C20	0.7860 (6)	0.20455 (18)	0.1007 (2)	0.0145 (9)	
C13	0.7848 (7)	0.31399 (18)	0.5103 (2)	0.0156 (9)	
H13A	0.823194	0.348621	0.501483	0.023*	
H13B	0.890648	0.296314	0.531695	0.023*	
H13C	0.677661	0.315238	0.533833	0.023*	
C27	0.7355 (6)	0.31943 (17)	−0.00920 (19)	0.0138 (9)	
H27A	0.688273	0.353526	−0.001780	0.021*	
H27B	0.638722	0.300470	−0.033070	0.021*	
H27C	0.849757	0.322077	−0.029428	0.021*	
C14	0.7197 (6)	0.22864 (17)	0.45570 (19)	0.0113 (8)	
C25	0.7812 (6)	0.23439 (17)	0.0480 (2)	0.0117 (8)	
C17	0.7117 (7)	0.12003 (18)	0.4666 (2)	0.0174 (10)	
H17	0.710667	0.084111	0.469778	0.021*	
C28	0.8313 (6)	0.37427 (16)	0.09623 (19)	0.0121 (9)	
H28A	0.702933	0.388882	0.095759	0.014*	
H28B	0.889213	0.386413	0.061739	0.014*	
C29	0.9539 (6)	0.39016 (17)	0.1520 (2)	0.0122 (9)	
H29	1.088626	0.380870	0.148407	0.015*	
C11	0.6906 (6)	0.36775 (17)	0.40348 (19)	0.0126 (9)	
H11A	0.822806	0.379269	0.402534	0.015*	
H11B	0.640320	0.382256	0.438117	0.015*	
C10	0.5701 (6)	0.38526 (17)	0.3486 (2)	0.0129 (9)	
H10	0.433277	0.380043	0.353554	0.015*	
C6	0.4719 (6)	0.50930 (18)	0.1912 (2)	0.0149 (9)	
C7	0.5306 (6)	0.49832 (17)	0.2530 (2)	0.0123 (9)	
C1	0.4262 (6)	0.47000 (17)	0.1482 (2)	0.0149 (9)	
C12	0.7266 (6)	0.28539 (17)	0.45387 (19)	0.0113 (8)	
C15	0.7586 (7)	0.20325 (18)	0.5103 (2)	0.0150 (9)	
H15	0.787888	0.223430	0.543726	0.018*	
C30	0.9411 (7)	0.44745 (17)	0.1646 (2)	0.0132 (9)	
H30A	1.022816	0.466698	0.140256	0.016*	
H30B	0.809436	0.459367	0.155977	0.016*	
C8	0.5798 (7)	0.54254 (18)	0.2947 (2)	0.0182 (10)	
H8A	0.633216	0.570576	0.273896	0.027*	
H8B	0.672434	0.531122	0.325863	0.027*	
H8C	0.465288	0.554100	0.311060	0.027*	
C31	1.0235 (6)	0.50243 (18)	0.2480 (2)	0.0150 (9)	
C22	0.7727 (7)	0.12579 (18)	0.0412 (2)	0.0197 (10)	
H22	0.769101	0.089808	0.039118	0.024*	
C23	0.7695 (7)	0.15510 (19)	−0.0106 (2)	0.0190 (10)	
H23	0.764424	0.138974	−0.047193	0.023*	
C24	0.7741 (7)	0.20798 (18)	−0.0062 (2)	0.0158 (9)	
H24	0.772348	0.227402	−0.040560	0.019*	
C9	0.6056 (7)	0.44167 (17)	0.33513 (19)	0.0136 (9)	
H9A	0.533894	0.463677	0.359735	0.016*	
H9B	0.741610	0.449663	0.343064	0.016*	
C38	1.1219 (7)	0.47204 (18)	0.3522 (2)	0.0171 (10)	
C33	1.0824 (6)	0.51236 (18)	0.3101 (2)	0.0156 (9)	
C5	0.4589 (7)	0.56179 (18)	0.1719 (2)	0.0189 (10)	
H5	0.490277	0.587974	0.199175	0.023*	
C40	0.2961 (8)	0.23976 (18)	0.1642 (2)	0.0206 (10)	
H40A	0.208940	0.259722	0.138055	0.031*	
H40B	0.224171	0.215968	0.185905	0.031*	
H40C	0.382332	0.220786	0.141722	0.031*	
C32	0.9831 (7)	0.54776 (18)	0.2068 (2)	0.0202 (10)	
H32A	0.930339	0.575865	0.227686	0.030*	
H32B	0.892338	0.537490	0.174626	0.030*	
H32C	1.100792	0.558715	0.191900	0.030*	
C34	1.1021 (7)	0.56421 (19)	0.3302 (2)	0.0227 (11)	
H34	1.074325	0.590881	0.303313	0.027*	
C4	0.4018 (8)	0.57527 (19)	0.1147 (2)	0.0243 (11)	
H4	0.392435	0.609970	0.103828	0.029*	
C18	0.6705 (7)	0.14284 (19)	0.4130 (2)	0.0196 (10)	
H18	0.641327	0.121833	0.380232	0.023*	
C2	0.3711 (7)	0.48541 (19)	0.0895 (2)	0.0211 (10)	
H2	0.342570	0.460076	0.061062	0.025*	
C3	0.3585 (8)	0.5367 (2)	0.0733 (2)	0.0236 (11)	
H3	0.320682	0.545600	0.034466	0.028*	
C21	0.7810 (7)	0.14995 (18)	0.0949 (2)	0.0194 (10)	
H21	0.783417	0.129738	0.128740	0.023*	
C42	1.1047 (8)	0.2371 (2)	0.3389 (2)	0.0256 (11)	
H42A	1.242630	0.238701	0.345497	0.038*	
H42B	1.067072	0.204078	0.322069	0.038*	
H42C	1.049827	0.241244	0.375637	0.038*	
C35	1.1599 (9)	0.5768 (2)	0.3872 (3)	0.0300 (13)	
H35	1.173262	0.611278	0.398564	0.036*	
C36	1.1985 (9)	0.5374 (2)	0.4282 (3)	0.0339 (14)	
H36	1.236433	0.545609	0.467253	0.041*	
C37	1.1808 (8)	0.4863 (2)	0.4112 (2)	0.0249 (11)	
H37	1.208207	0.460476	0.439069	0.030*	

### Atomic displacement parameters (Å^2^)

**Table:**

	*U*^11^	*U*^22^	*U*^33^	*U*^12^	*U*^13^	*U*^23^
Cu2	0.0193 (3)	0.0094 (3)	0.0074 (3)	0.0025 (2)	−0.0014 (2)	−0.0003 (2)
Cu1	0.0176 (3)	0.0091 (3)	0.0087 (3)	−0.0023 (2)	−0.0022 (2)	0.0013 (2)
Cu3	0.0198 (3)	0.0098 (3)	0.0073 (3)	−0.0045 (2)	−0.0008 (2)	0.0007 (2)
Cu4	0.0161 (3)	0.0101 (3)	0.0100 (3)	0.0023 (2)	−0.0029 (2)	−0.0027 (2)
O5	0.0194 (16)	0.0129 (16)	0.0070 (15)	−0.0049 (13)	0.0006 (12)	−0.0013 (12)
O2	0.0222 (17)	0.0105 (15)	0.0091 (15)	0.0017 (12)	−0.0001 (12)	−0.0017 (12)
O10	0.040 (2)	0.0114 (17)	0.0146 (17)	−0.0102 (15)	0.0000 (15)	0.0005 (13)
O7	0.0219 (17)	0.0133 (17)	0.0176 (18)	0.0033 (13)	−0.0062 (13)	−0.0013 (13)
O3	0.0324 (19)	0.0102 (16)	0.0123 (16)	0.0036 (14)	−0.0016 (14)	−0.0016 (13)
O4	0.0315 (19)	0.0108 (16)	0.0113 (16)	−0.0033 (13)	−0.0014 (14)	0.0001 (13)
O1	0.0227 (17)	0.0093 (15)	0.0125 (17)	−0.0023 (12)	−0.0040 (13)	0.0029 (12)
O6	0.0260 (18)	0.0109 (16)	0.0158 (18)	0.0015 (13)	−0.0054 (14)	−0.0052 (13)
O11	0.0298 (19)	0.0160 (17)	0.0137 (17)	−0.0020 (14)	−0.0034 (14)	0.0002 (13)
O8	0.0314 (19)	0.0184 (18)	0.0114 (17)	−0.0083 (14)	−0.0038 (14)	0.0047 (13)
N1	0.0165 (18)	0.0117 (19)	0.0093 (19)	0.0018 (14)	−0.0001 (15)	0.0004 (14)
C41	0.020 (2)	0.012 (2)	0.008 (2)	−0.0020 (17)	0.0000 (17)	0.0008 (17)
N3	0.0141 (18)	0.0114 (18)	0.0076 (18)	−0.0008 (14)	0.0005 (14)	0.0007 (14)
O9	0.053 (2)	0.0092 (16)	0.0194 (19)	−0.0017 (16)	0.0144 (17)	−0.0007 (14)
N2	0.0147 (18)	0.0110 (18)	0.0086 (18)	−0.0016 (14)	0.0015 (14)	−0.0024 (14)
N4	0.0136 (18)	0.0144 (19)	0.0080 (18)	0.0009 (14)	−0.0006 (14)	−0.0019 (14)
C39	0.017 (2)	0.014 (2)	0.012 (2)	0.0041 (17)	0.0022 (17)	−0.0051 (18)
C19	0.016 (2)	0.015 (2)	0.009 (2)	0.0034 (17)	0.0010 (17)	0.0008 (17)
C26	0.0094 (19)	0.014 (2)	0.009 (2)	0.0010 (16)	0.0019 (16)	0.0022 (17)
C16	0.023 (2)	0.018 (2)	0.013 (2)	0.0036 (19)	0.0018 (19)	0.0033 (18)
C20	0.016 (2)	0.014 (2)	0.013 (2)	−0.0024 (17)	−0.0026 (17)	−0.0024 (17)
C13	0.021 (2)	0.015 (2)	0.010 (2)	−0.0007 (18)	−0.0033 (18)	−0.0007 (17)
C27	0.019 (2)	0.012 (2)	0.010 (2)	−0.0004 (17)	−0.0016 (17)	0.0010 (17)
C14	0.013 (2)	0.011 (2)	0.010 (2)	0.0027 (16)	0.0008 (16)	0.0011 (16)
C25	0.011 (2)	0.010 (2)	0.013 (2)	−0.0013 (16)	−0.0007 (16)	0.0006 (17)
C17	0.026 (2)	0.011 (2)	0.016 (2)	0.0021 (18)	0.0032 (19)	0.0027 (18)
C28	0.018 (2)	0.009 (2)	0.009 (2)	0.0009 (16)	0.0009 (17)	0.0005 (16)
C29	0.013 (2)	0.010 (2)	0.013 (2)	0.0006 (16)	0.0026 (17)	0.0008 (17)
C11	0.019 (2)	0.012 (2)	0.006 (2)	−0.0015 (17)	−0.0012 (17)	−0.0015 (16)
C10	0.015 (2)	0.011 (2)	0.013 (2)	0.0004 (16)	0.0002 (17)	−0.0010 (17)
C6	0.016 (2)	0.013 (2)	0.015 (2)	−0.0029 (17)	0.0019 (18)	0.0025 (18)
C7	0.011 (2)	0.011 (2)	0.015 (2)	−0.0008 (16)	0.0027 (17)	−0.0006 (17)
C1	0.015 (2)	0.011 (2)	0.019 (2)	−0.0008 (17)	0.0009 (18)	0.0035 (18)
C12	0.0106 (19)	0.012 (2)	0.011 (2)	−0.0005 (16)	0.0024 (16)	−0.0007 (17)
C15	0.018 (2)	0.017 (2)	0.009 (2)	0.0005 (17)	0.0011 (17)	0.0002 (18)
C30	0.018 (2)	0.013 (2)	0.009 (2)	−0.0024 (17)	0.0006 (17)	−0.0027 (17)
C8	0.024 (2)	0.012 (2)	0.017 (2)	−0.0014 (18)	−0.0004 (19)	−0.0027 (19)
C31	0.011 (2)	0.013 (2)	0.021 (3)	0.0017 (17)	0.0033 (17)	−0.0001 (18)
C22	0.027 (3)	0.010 (2)	0.022 (3)	−0.0043 (18)	0.004 (2)	−0.0017 (19)
C23	0.024 (2)	0.018 (2)	0.016 (2)	−0.0026 (19)	0.0020 (19)	−0.0049 (19)
C24	0.018 (2)	0.018 (2)	0.011 (2)	−0.0009 (18)	0.0012 (17)	0.0015 (18)
C9	0.018 (2)	0.014 (2)	0.009 (2)	0.0018 (17)	0.0029 (17)	0.0004 (17)
C38	0.016 (2)	0.016 (2)	0.019 (2)	0.0026 (17)	−0.0010 (18)	−0.0039 (19)
C33	0.016 (2)	0.013 (2)	0.018 (2)	0.0001 (17)	0.0012 (18)	−0.0063 (18)
C5	0.024 (2)	0.012 (2)	0.021 (3)	−0.0036 (18)	0.004 (2)	0.0026 (19)
C40	0.032 (3)	0.013 (2)	0.016 (2)	−0.003 (2)	−0.002 (2)	0.0004 (19)
C32	0.026 (3)	0.013 (2)	0.021 (3)	−0.0004 (19)	−0.001 (2)	0.0007 (19)
C34	0.025 (3)	0.016 (2)	0.026 (3)	0.003 (2)	0.002 (2)	−0.005 (2)
C4	0.036 (3)	0.012 (2)	0.024 (3)	−0.001 (2)	0.004 (2)	0.008 (2)
C18	0.027 (3)	0.016 (2)	0.015 (2)	0.0018 (19)	0.0003 (19)	−0.0048 (19)
C2	0.029 (3)	0.015 (2)	0.019 (3)	−0.004 (2)	−0.004 (2)	0.0023 (19)
C3	0.029 (3)	0.021 (3)	0.019 (3)	−0.001 (2)	−0.005 (2)	0.011 (2)
C21	0.028 (3)	0.013 (2)	0.016 (2)	−0.0007 (19)	−0.003 (2)	0.0024 (18)
C42	0.033 (3)	0.021 (3)	0.021 (3)	−0.002 (2)	−0.008 (2)	0.008 (2)
C35	0.044 (3)	0.017 (3)	0.028 (3)	0.003 (2)	−0.003 (2)	−0.013 (2)
C36	0.052 (4)	0.028 (3)	0.019 (3)	0.002 (3)	−0.007 (3)	−0.015 (2)
C37	0.035 (3)	0.020 (3)	0.018 (3)	0.003 (2)	−0.004 (2)	−0.006 (2)

### Geometric parameters (Å, º)

**Table:**

Cu2—O2	1.920 (3)	C17—C18	1.364 (7)
Cu2—O3	1.877 (3)	C28—H28A	0.9700
Cu2—O11	1.940 (3)	C28—H28B	0.9700
Cu2—O8	2.703 (4)	C28—C29	1.522 (6)
Cu2—N2	1.961 (4)	C29—H29	0.9800
Cu1—O5	2.749 (3)	C29—C30	1.510 (6)
Cu1—O2	1.916 (3)	C11—H11A	0.9700
Cu1—O10	1.982 (3)	C11—H11B	0.9700
Cu1—O1	1.878 (3)	C11—C10	1.509 (6)
Cu1—N1	1.966 (4)	C10—H10	0.9800
Cu3—O5	1.907 (3)	C10—C9	1.513 (6)
Cu3—O4	1.873 (3)	C6—C7	1.458 (6)
Cu3—O8	1.957 (3)	C6—C1	1.427 (7)
Cu3—N3	1.947 (4)	C6—C5	1.424 (6)
Cu4—O5	1.921 (3)	C7—C8	1.506 (6)
Cu4—O7	1.955 (3)	C1—C2	1.415 (7)
Cu4—O6	1.869 (3)	C15—H15	0.9300
Cu4—N4	1.962 (4)	C30—H30A	0.9700
O5—C29	1.424 (5)	C30—H30B	0.9700
O2—C10	1.432 (5)	C8—H8A	0.9600
O10—C39	1.244 (6)	C8—H8B	0.9600
O7—C41	1.251 (5)	C8—H8C	0.9600
O3—C19	1.313 (5)	C31—C33	1.461 (7)
O4—C20	1.319 (5)	C31—C32	1.511 (7)
O1—C1	1.317 (5)	C22—H22	0.9300
O6—C38	1.318 (6)	C22—C23	1.403 (7)
O11—C39	1.248 (6)	C22—C21	1.373 (7)
O8—C41	1.266 (6)	C23—H23	0.9300
N1—C7	1.300 (6)	C23—C24	1.369 (7)
N1—C9	1.472 (6)	C24—H24	0.9300
C41—C42	1.503 (6)	C9—H9A	0.9700
N3—C26	1.291 (6)	C9—H9B	0.9700
N3—C28	1.474 (5)	C38—C33	1.426 (7)
O9—H9C	0.8499	C38—C37	1.418 (7)
O9—H9D	0.8500	C33—C34	1.417 (6)
N2—C11	1.476 (6)	C5—H5	0.9300
N2—C12	1.297 (6)	C5—C4	1.374 (7)
N4—C30	1.467 (5)	C40—H40A	0.9600
N4—C31	1.299 (6)	C40—H40B	0.9600
C39—C40	1.520 (6)	C40—H40C	0.9600
C19—C14	1.424 (6)	C32—H32A	0.9600
C19—C18	1.414 (7)	C32—H32B	0.9600
C26—C27	1.512 (6)	C32—H32C	0.9600
C26—C25	1.472 (6)	C34—H34	0.9300
C16—H16	0.9300	C34—C35	1.366 (7)
C16—C17	1.399 (7)	C4—H4	0.9300
C16—C15	1.371 (7)	C4—C3	1.385 (8)
C20—C25	1.427 (6)	C18—H18	0.9300
C20—C21	1.415 (6)	C2—H2	0.9300
C13—H13A	0.9600	C2—C3	1.376 (7)
C13—H13B	0.9600	C3—H3	0.9300
C13—H13C	0.9600	C21—H21	0.9300
C13—C12	1.508 (6)	C42—H42A	0.9600
C27—H27A	0.9600	C42—H42B	0.9600
C27—H27B	0.9600	C42—H42C	0.9600
C27—H27C	0.9600	C35—H35	0.9300
C14—C12	1.466 (6)	C35—C36	1.389 (8)
C14—C15	1.412 (6)	C36—H36	0.9300
C25—C24	1.409 (6)	C36—C37	1.377 (7)
C17—H17	0.9300	C37—H37	0.9300
			
O2—Cu2—O11	96.89 (14)	C30—C29—C28	112.6 (4)
O2—Cu2—O8	84.89 (12)	C30—C29—H29	109.3
O2—Cu2—N2	85.74 (14)	N2—C11—H11A	110.2
O3—Cu2—O2	173.00 (15)	N2—C11—H11B	110.2
O3—Cu2—O11	86.73 (14)	N2—C11—C10	107.7 (3)
O3—Cu2—O8	89.68 (13)	H11A—C11—H11B	108.5
O3—Cu2—N2	92.70 (15)	C10—C11—H11A	110.2
O11—Cu2—O8	82.40 (13)	C10—C11—H11B	110.2
O11—Cu2—N2	161.66 (15)	O2—C10—C11	107.7 (4)
N2—Cu2—O8	115.93 (13)	O2—C10—H10	109.6
O2—Cu1—O5	80.48 (12)	O2—C10—C9	108.7 (4)
O2—Cu1—O10	95.10 (14)	C11—C10—H10	109.6
O2—Cu1—N1	85.01 (14)	C11—C10—C9	111.6 (4)
O10—Cu1—O5	83.75 (13)	C9—C10—H10	109.6
O1—Cu1—O5	97.68 (12)	C1—C6—C7	123.5 (4)
O1—Cu1—O2	176.33 (14)	C5—C6—C7	119.2 (4)
O1—Cu1—O10	87.83 (14)	C5—C6—C1	117.4 (4)
O1—Cu1—N1	92.51 (15)	N1—C7—C6	121.3 (4)
N1—Cu1—O5	106.38 (13)	N1—C7—C8	119.3 (4)
N1—Cu1—O10	169.71 (16)	C6—C7—C8	119.4 (4)
O5—Cu3—O8	92.44 (14)	O1—C1—C6	125.6 (4)
O5—Cu3—N3	86.17 (14)	O1—C1—C2	116.1 (4)
O4—Cu3—O5	177.07 (15)	C2—C1—C6	118.3 (4)
O4—Cu3—O8	88.43 (14)	N2—C12—C13	120.5 (4)
O4—Cu3—N3	93.29 (15)	N2—C12—C14	121.3 (4)
N3—Cu3—O8	173.28 (15)	C14—C12—C13	118.1 (4)
O5—Cu4—O7	96.93 (14)	C16—C15—C14	123.5 (4)
O5—Cu4—N4	84.04 (14)	C16—C15—H15	118.2
O7—Cu4—N4	164.11 (15)	C14—C15—H15	118.2
O6—Cu4—O5	170.48 (14)	N4—C30—C29	108.0 (4)
O6—Cu4—O7	87.96 (14)	N4—C30—H30A	110.1
O6—Cu4—N4	93.50 (15)	N4—C30—H30B	110.1
Cu3—O5—Cu1	98.74 (12)	C29—C30—H30A	110.1
Cu3—O5—Cu4	129.24 (17)	C29—C30—H30B	110.1
Cu4—O5—Cu1	95.83 (12)	H30A—C30—H30B	108.4
C29—O5—Cu1	116.7 (2)	C7—C8—H8A	109.5
C29—O5—Cu3	108.9 (3)	C7—C8—H8B	109.5
C29—O5—Cu4	107.0 (2)	C7—C8—H8C	109.5
Cu1—O2—Cu2	129.60 (17)	H8A—C8—H8B	109.5
C10—O2—Cu2	105.8 (3)	H8A—C8—H8C	109.5
C10—O2—Cu1	108.9 (3)	H8B—C8—H8C	109.5
C39—O10—Cu1	132.3 (3)	N4—C31—C33	122.0 (4)
C41—O7—Cu4	129.8 (3)	N4—C31—C32	118.8 (4)
C19—O3—Cu2	128.0 (3)	C33—C31—C32	119.2 (4)
C20—O4—Cu3	126.4 (3)	C23—C22—H22	119.8
C1—O1—Cu1	127.5 (3)	C21—C22—H22	119.8
C38—O6—Cu4	126.8 (3)	C21—C22—C23	120.3 (4)
C39—O11—Cu2	133.4 (3)	C22—C23—H23	120.8
Cu3—O8—Cu2	113.47 (15)	C24—C23—C22	118.5 (5)
C41—O8—Cu2	95.5 (3)	C24—C23—H23	120.8
C41—O8—Cu3	130.7 (3)	C25—C24—H24	118.4
C7—N1—Cu1	128.8 (3)	C23—C24—C25	123.1 (4)
C7—N1—C9	119.5 (4)	C23—C24—H24	118.4
C9—N1—Cu1	111.1 (3)	N1—C9—C10	108.4 (4)
O7—C41—O8	125.8 (4)	N1—C9—H9A	110.0
O7—C41—C42	118.0 (4)	N1—C9—H9B	110.0
O8—C41—C42	116.1 (4)	C10—C9—H9A	110.0
C26—N3—Cu3	128.5 (3)	C10—C9—H9B	110.0
C26—N3—C28	121.0 (4)	H9A—C9—H9B	108.4
C28—N3—Cu3	110.0 (3)	O6—C38—C33	126.0 (4)
H9C—O9—H9D	104.5	O6—C38—C37	115.9 (4)
C11—N2—Cu2	109.6 (3)	C37—C38—C33	118.1 (4)
C12—N2—Cu2	129.1 (3)	C38—C33—C31	123.0 (4)
C12—N2—C11	121.3 (4)	C34—C33—C31	119.3 (4)
C30—N4—Cu4	111.4 (3)	C34—C33—C38	117.6 (5)
C31—N4—Cu4	127.9 (3)	C6—C5—H5	118.7
C31—N4—C30	120.3 (4)	C4—C5—C6	122.6 (5)
O10—C39—O11	127.6 (4)	C4—C5—H5	118.7
O10—C39—C40	117.2 (4)	C39—C40—H40A	109.5
O11—C39—C40	115.2 (4)	C39—C40—H40B	109.5
O3—C19—C14	125.2 (4)	C39—C40—H40C	109.5
O3—C19—C18	116.8 (4)	H40A—C40—H40B	109.5
C18—C19—C14	117.9 (4)	H40A—C40—H40C	109.5
N3—C26—C27	120.4 (4)	H40B—C40—H40C	109.5
N3—C26—C25	121.6 (4)	C31—C32—H32A	109.5
C25—C26—C27	118.0 (4)	C31—C32—H32B	109.5
C17—C16—H16	120.8	C31—C32—H32C	109.5
C15—C16—H16	120.8	H32A—C32—H32B	109.5
C15—C16—C17	118.3 (4)	H32A—C32—H32C	109.5
O4—C20—C25	125.8 (4)	H32B—C32—H32C	109.5
O4—C20—C21	116.8 (4)	C33—C34—H34	118.5
C21—C20—C25	117.4 (4)	C35—C34—C33	122.9 (5)
H13A—C13—H13B	109.5	C35—C34—H34	118.5
H13A—C13—H13C	109.5	C5—C4—H4	120.3
H13B—C13—H13C	109.5	C5—C4—C3	119.5 (5)
C12—C13—H13A	109.5	C3—C4—H4	120.3
C12—C13—H13B	109.5	C19—C18—H18	118.8
C12—C13—H13C	109.5	C17—C18—C19	122.5 (4)
C26—C27—H27A	109.5	C17—C18—H18	118.8
C26—C27—H27B	109.5	C1—C2—H2	119.0
C26—C27—H27C	109.5	C3—C2—C1	122.0 (5)
H27A—C27—H27B	109.5	C3—C2—H2	119.0
H27A—C27—H27C	109.5	C4—C3—H3	119.9
H27B—C27—H27C	109.5	C2—C3—C4	120.2 (5)
C19—C14—C12	123.5 (4)	C2—C3—H3	119.9
C15—C14—C19	117.5 (4)	C20—C21—H21	118.9
C15—C14—C12	119.0 (4)	C22—C21—C20	122.2 (5)
C20—C25—C26	122.2 (4)	C22—C21—H21	118.9
C24—C25—C26	119.4 (4)	C41—C42—H42A	109.5
C24—C25—C20	118.4 (4)	C41—C42—H42B	109.5
C16—C17—H17	119.9	C41—C42—H42C	109.5
C18—C17—C16	120.3 (4)	H42A—C42—H42B	109.5
C18—C17—H17	119.9	H42A—C42—H42C	109.5
N3—C28—H28A	110.4	H42B—C42—H42C	109.5
N3—C28—H28B	110.4	C34—C35—H35	120.3
N3—C28—C29	106.5 (3)	C34—C35—C36	119.3 (5)
H28A—C28—H28B	108.6	C36—C35—H35	120.3
C29—C28—H28A	110.4	C35—C36—H36	119.9
C29—C28—H28B	110.4	C37—C36—C35	120.2 (5)
O5—C29—C28	108.0 (3)	C37—C36—H36	119.9
O5—C29—H29	109.3	C38—C37—H37	119.1
O5—C29—C30	108.4 (4)	C36—C37—C38	121.8 (5)
C28—C29—H29	109.3	C36—C37—H37	119.1
			
Cu2—O2—C10—C11	50.9 (4)	N2—C11—C10—O2	−47.4 (5)
Cu2—O2—C10—C9	172.0 (3)	N2—C11—C10—C9	−166.7 (4)
Cu2—O3—C19—C14	−2.5 (7)	N4—Cu4—O6—C38	7.3 (4)
Cu2—O3—C19—C18	178.5 (3)	N4—C31—C33—C38	−0.5 (7)
Cu2—O11—C39—O10	−4.0 (8)	N4—C31—C33—C34	179.5 (4)
Cu2—O11—C39—C40	175.8 (3)	C19—C14—C12—N2	1.8 (7)
Cu2—O8—C41—O7	−90.7 (5)	C19—C14—C12—C13	−178.8 (4)
Cu2—O8—C41—C42	85.7 (4)	C19—C14—C15—C16	1.5 (7)
Cu2—N2—C11—C10	21.0 (4)	C26—N3—C28—C29	−158.3 (4)
Cu2—N2—C12—C13	178.5 (3)	C26—C25—C24—C23	−178.9 (4)
Cu2—N2—C12—C14	−2.0 (6)	C16—C17—C18—C19	0.2 (8)
Cu1—O5—C29—C28	−66.2 (4)	C20—C25—C24—C23	0.7 (7)
Cu1—O5—C29—C30	56.0 (4)	C27—C26—C25—C20	−167.5 (4)
Cu1—O2—C10—C11	−166.2 (3)	C27—C26—C25—C24	12.0 (6)
Cu1—O2—C10—C9	−45.1 (4)	C14—C19—C18—C17	1.3 (7)
Cu1—O10—C39—O11	−22.5 (8)	C25—C20—C21—C22	0.3 (7)
Cu1—O10—C39—C40	157.7 (4)	C17—C16—C15—C14	0.1 (7)
Cu1—O1—C1—C6	−4.2 (7)	C28—N3—C26—C27	−2.4 (6)
Cu1—O1—C1—C2	174.4 (3)	C28—N3—C26—C25	177.3 (4)
Cu1—N1—C7—C6	8.2 (6)	C28—C29—C30—N4	160.4 (4)
Cu1—N1—C7—C8	−171.3 (3)	C11—N2—C12—C13	0.8 (6)
Cu1—N1—C9—C10	−18.5 (4)	C11—N2—C12—C14	−179.7 (4)
Cu3—O5—C29—C28	44.4 (4)	C11—C10—C9—N1	159.8 (4)
Cu3—O5—C29—C30	166.7 (3)	C6—C1—C2—C3	−0.8 (7)
Cu3—O4—C20—C25	−14.1 (7)	C6—C5—C4—C3	−1.3 (8)
Cu3—O4—C20—C21	167.4 (3)	C7—N1—C9—C10	169.2 (4)
Cu3—O8—C41—O7	36.9 (7)	C7—C6—C1—O1	−1.4 (7)
Cu3—O8—C41—C42	−146.7 (4)	C7—C6—C1—C2	−179.9 (4)
Cu3—N3—C26—C27	168.8 (3)	C7—C6—C5—C4	−179.0 (5)
Cu3—N3—C26—C25	−11.5 (6)	C1—C6—C7—N1	−0.8 (7)
Cu3—N3—C28—C29	29.0 (4)	C1—C6—C7—C8	178.7 (4)
Cu4—O5—C29—C28	−172.1 (3)	C1—C6—C5—C4	1.1 (7)
Cu4—O5—C29—C30	−49.8 (4)	C1—C2—C3—C4	0.6 (8)
Cu4—O7—C41—O8	7.7 (7)	C12—N2—C11—C10	−160.9 (4)
Cu4—O7—C41—C42	−168.7 (3)	C12—C14—C15—C16	179.9 (4)
Cu4—O6—C38—C33	−3.7 (7)	C15—C16—C17—C18	−1.0 (7)
Cu4—O6—C38—C37	175.7 (3)	C15—C14—C12—N2	−176.6 (4)
Cu4—N4—C30—C29	−13.5 (4)	C15—C14—C12—C13	2.9 (6)
Cu4—N4—C31—C33	7.3 (6)	C30—N4—C31—C33	178.8 (4)
Cu4—N4—C31—C32	−172.6 (3)	C30—N4—C31—C32	−1.1 (6)
O5—Cu1—O1—C1	−98.9 (4)	C31—N4—C30—C29	173.8 (4)
O5—C29—C30—N4	41.0 (5)	C31—C33—C34—C35	−178.8 (5)
O2—C10—C9—N1	41.1 (5)	C22—C23—C24—C25	−0.2 (7)
O10—Cu1—O1—C1	177.7 (4)	C23—C22—C21—C20	0.2 (8)
O7—Cu4—O6—C38	171.5 (4)	C9—N1—C7—C6	179.0 (4)
O3—C19—C14—C12	0.6 (7)	C9—N1—C7—C8	−0.5 (6)
O3—C19—C14—C15	178.9 (4)	C38—C33—C34—C35	1.3 (8)
O3—C19—C18—C17	−179.6 (4)	C33—C38—C37—C36	0.6 (8)
O4—C20—C25—C26	0.4 (7)	C33—C34—C35—C36	−1.2 (9)
O4—C20—C25—C24	−179.2 (4)	C5—C6—C7—N1	179.4 (4)
O4—C20—C21—C22	178.9 (4)	C5—C6—C7—C8	−1.2 (6)
O1—C1—C2—C3	−179.4 (5)	C5—C6—C1—O1	178.5 (4)
O6—C38—C33—C31	−1.5 (7)	C5—C6—C1—C2	0.0 (7)
O6—C38—C33—C34	178.4 (4)	C5—C4—C3—C2	0.5 (8)
O6—C38—C37—C36	−178.8 (5)	C32—C31—C33—C38	179.4 (4)
O11—Cu2—O3—C19	−159.8 (4)	C32—C31—C33—C34	−0.5 (7)
O8—Cu2—O3—C19	117.8 (4)	C34—C35—C36—C37	0.8 (9)
O8—Cu3—O4—C20	−161.1 (4)	C18—C19—C14—C12	179.6 (4)
N1—Cu1—O1—C1	8.0 (4)	C18—C19—C14—C15	−2.1 (6)
N3—Cu3—O4—C20	12.3 (4)	C21—C20—C25—C26	178.9 (4)
N3—C26—C25—C20	12.8 (6)	C21—C20—C25—C24	−0.7 (6)
N3—C26—C25—C24	−167.7 (4)	C21—C22—C23—C24	−0.3 (7)
N3—C28—C29—O5	−47.9 (4)	C35—C36—C37—C38	−0.5 (9)
N3—C28—C29—C30	−167.5 (4)	C37—C38—C33—C31	179.1 (4)
N2—Cu2—O3—C19	1.9 (4)	C37—C38—C33—C34	−0.9 (7)

### Hydrogen-bond geometry (Å, º)

**Table:**

*D*—H···*A*	*D*—H	H···*A*	*D*···*A*	*D*—H···*A*
O9—H9*C*···O4	0.85	2.08	2.894 (5)	159
O9—H9*C*···O8	0.85	2.56	3.158 (5)	128
O9—H9*D*···O3	0.85	2.08	2.928 (5)	175
C28—H28*A*···O1	0.97	2.58	3.427 (6)	146
C29—H29···O1^i^	0.98	2.60	3.424 (5)	142
C10—H10···O6^ii^	0.98	2.51	3.351 (6)	144
C8—H8*A*···O9^iii^	0.96	2.44	3.372 (6)	163
C9—H9*B*···O6	0.97	2.65	3.521 (6)	150
C32—H32*A*···O9^iii^	0.96	2.38	3.304 (6)	162
C42—H42*A*···O11^i^	0.96	2.66	3.256 (7)	121

Symmetry codes: (i) *x*+1, *y*, *z*; (ii) *x*−1, *y*, *z*; (iii) −*x*+3/2, *y*+1/2, −*z*+1/2.

## References

[bb1] Addison, A. W., Rao, T. N., Reedijk, J., van Rijn, J. & Verschoor, G. C. (1984). *J. Chem. Soc. Dalton Trans.* pp. 1349–1356.

[bb2] Aly, M. M. (1999). *J. Coord. Chem.* **47**, 505–521.

[bb3] Asadi, Z., Golchin, M., Eigner, V., Dusek, M. & Amirghofran, Z. (2018). *J. Photochem. Photobiol. Chem.* **361**, 93–104.

[bb4] Banerjee, A. & Chattopadhyay, S. (2019). *Polyhedron*, **159**, 1–11.

[bb5] Banerjee, S., Nandy, M., Sen, S., Mandal, S., Rosair, G. M., Slawin, A. M. Z., Gómez García, C. J., Clemente-Juan, J. M., Zangrando, E., Guidolin, N. & Mitra, S. (2011). *Dalton Trans.* **40**, 1652–1661. 10.1039/c0dt00923g 21243136

[bb6] Basak, S., Sen, S., Rosair, G., Desplanches, C., Garribba, E. & Mitra, S. (2009). *Aust. J. Chem.* **62**, 366–375.

[bb7] Biswas, R., Diaz, C., Bauzá, A., Frontera, A. & Ghosh, A. (2013). *Dalton Trans.* **42**, 12274–12283. 10.1039/c3dt51153g 23846248

[bb8] Bruker (2016). *APEX3* and *SAINT*. Bruker AXS Inc., Madison, Wisconsin, USA.

[bb9] Das, A., Goswami, S. & Ghosh, A. (2018). *New J. Chem.* **42**, 19377–19389.

[bb10] Dolomanov, O. V., Bourhis, L. J., Gildea, R. J., Howard, J. A. K. & Puschmann, H. (2009). *J. Appl. Cryst.* **42**, 339–341.

[bb11] Ferguson, A., Parkin, A. & Murrie, M. (2006). *Dalton Trans.* pp. 3627–3628. 10.1039/b603622h 16865173

[bb12] Fernandes, C., Neves, A., Vencato, I., Bortoluzzi, A. J., Drago, V., Weyhermüller, T. & Rentschler, E. (2000). *Chem. Lett.* **29**, 540–541.

[bb13] Gheorghe, R., Ionita, G. A., Maxim, C., Caneschi, A., Sorace, L. & Andruh, M. (2019). *Polyhedron*, **171**, 269–278.

[bb14] Ghosh, A. K., Bauzá, A., Bertolasi, V., Frontera, A. & Ray, D. (2013). *Polyhedron*, **53**, 32–39.

[bb15] Ghosh, A. K., Clérac, R., Mathonière, C. & Ray, D. (2013). *Polyhedron*, **54**, 196–200.

[bb42] Groom, C. R., Bruno, I. J., Lightfoot, M. P. & Ward, S. C. (2016). *Acta Cryst*. B**72**, 171–179. 10.1107/S2052520616003954 PMC4822653 27048719

[bb16] Haldar, S., Patra, A., Vijaykumar, G., Carrella, L. & Bera, M. (2016). *Polyhedron*, **117**, 542–551.

[bb17] Hari, N., Ghosh, S. & Mohanta, S. (2019). *Inorg. Chim. Acta*, **491**, 34–41.

[bb18] Kébé, M., Thiam, I. E., Sow, M. M., Diouf, O., Barry, A. H., Sall, A. S., Retailleau, P. & Gaye, M. (2021). *Acta Cryst.* E**77**, 708–713. 10.1107/S2056989021005570 PMC8382066 34513016

[bb19] Krause, L., Herbst-Irmer, R., Sheldrick, G. M. & Stalke, D. (2015). *J. Appl. Cryst.* **48**, 3–10. 10.1107/S1600576714022985 PMC4453166 26089746

[bb20] Liu, S., Wang, S., Cao, F., Fu, H., Li, D. & Dou, J. (2012). *RSC Adv.* **2**, 1310–1313.

[bb21] Lukov, V. V., Shcherbakov, I. N., Levchenkov, S. I., Popov, L. D. & Pankov, I. V. (2017). *Russ. J. Coord. Chem.* **43**, 1–20.

[bb22] Mamour, S., Mayoro, D., Elhadj Ibrahima, T., Mohamed, G., Aliou Hamady, B. & Ellena, J. (2018). *Acta Cryst.* E**74**, 642–645. 10.1107/S2056989018005261 PMC5947478 29850082

[bb23] Manna, S., Zangrando, E., Puschmann, H. & Manna, S. C. (2019). *Polyhedron*, **162**, 285–292.

[bb24] Maurya, M. R., Bisht, M., Chaudhary, N., Avecilla, F., Kumar, U. & Hsu, H.-F. (2013). *Polyhedron*, **54**, 180–188.

[bb25] Mitra, M., Maji, A. K., Ghosh, B. K., Raghavaiah, P., Ribas, J. & Ghosh, R. (2014). *Polyhedron*, **67**, 19–26.

[bb26] Monfared, H. H., Sanchiz, J., Kalantari, Z. & Janiak, C. (2009). *Inorg. Chim. Acta*, **362**, 3791–3795.

[bb27] Nesterova, O. V., Bondarenko, O. E., Pombeiro, A. J. L. & Nesterov, D. S. (2020). *Dalton Trans.* **49**, 4710–4724. 10.1039/d0dt00222d 32207490

[bb28] Osypiuk, D., Cristóvão, B. & Bartyzel, A. (2020). *Crystals*, **10**, 1004.

[bb29] Patra, A., Haldar, S., Kumar, G. V., Carrella, L., Ghosh, A. K. & Bera, M. (2015). *Inorg. Chim. Acta*, **436**, 195–204.

[bb30] Popov, L. D., Levchenkov, S. I., Shcherbakov, I. N., Lukov, V. V., Suponitsky, K. Y. & Kogan, V. A. (2012). *Inorg. Chem. Commun.* **17**, 1–4.

[bb31] Sall, O., Tamboura, F. B., Sy, A., Barry, A. H., Thiam, E. I., Gaye, M. & Ellena, J. (2019). *Acta Cryst.* E**75**, 1069–1075. 10.1107/S2056989019008922 PMC6659337 31392027

[bb32] Sanyal, R., Ketkov, S., Purkait, S., Mautner, F. A., Zhigulin, G. & Das, D. (2017). *New J. Chem.* **41**, 8586–8597.

[bb33] Sarr, M., Diop, M., Thiam, I. E., Gaye, M., Barry, A. H., Alvarez, N. & Ellena, J. (2018*a*). *Eur. J. Chem.* **9**, 67–73.

[bb34] Sarr, M., Diop, M., Thiam, E. I., Gaye, M., Barry, A. H., Orton, J. B. & Coles, S. J. (2018*b*). *Acta Cryst.* E**74**, 1862–1866. 10.1107/S2056989018016109 PMC6281091 30574389

[bb35] Sheldrick, G. M. (2015*a*). *Acta Cryst.* A**71**, 3–8.

[bb36] Sheldrick, G. M. (2015*b*). *Acta Cryst.* C**71**, 3–8.

[bb37] Shit, S., Nandy, M., Rosair, G., Fallah, M. S. E., Ribas, J., Garribba, E. & Mitra, S. (2013). *Polyhedron*, **52**, 963–969.

[bb38] Siluvai, G. S. & Murthy, N. N. (2009). *Polyhedron*, **28**, 2149–2156.

[bb39] Singh, Y. P., Patel, R. N., Singh, Y., Choquesillo-Lazarte, D. & Butcher, R. J. (2017). *Dalton Trans.* **46**, 2803–2820. 10.1039/c6dt04661d 28174782

[bb41] Song, Y., Gamez, P., Roubeau, O., Lutz, M., Spek, A. L. & Reedijk, J. (2003). *Eur. J. Inorg. Chem*. pp. 2924–2928.

[bb40] Xie, Q.-W., Chen, X., Hu, K.-Q., Wang, Y.-T., Cui, A.-L. & Kou, H.-Z. (2012). *Polyhedron*, **38**, 213–217.

